# Antibiotic dispensing practices in community pharmacies: Implications for antimicrobial stewardship in resource-constrained settings

**DOI:** 10.1016/j.rcsop.2025.100606

**Published:** 2025-04-22

**Authors:** Abdullah Al Masud, Ramesh Lahiru Walpola, Malabika Sarker, Muhammad Asaduzzaman, Md. Saiful Islam, Ayesha Tasnim Mostafa, Zubair Akhtar, Alamgir Kabir, Holly Seale

**Affiliations:** a School of Population Health, Faculty of Medicine and Health, University of New South Wales, Sydney, Australia; b School of Health Sciences, Faculty of Medicine and Health, University of New South Wales, Sydney, Australia; c The George Institute for Global Health, University of New South Wales, Sydney, Australia; d Brown School of Public Health, Brown University, USA; e Heidelberg Institute of Global Health, Heidelberg University, Germany; f Department of Community Medicine and Global Health, Institute of Health and Society, Faculty of Medicine, University of Oslo, Oslo, Norway; g BRAC James P. Grant School of Public Health, BRAC University, Dhaka, Bangladesh; h The Kirby Institute, Faculty of Medicine and Health, University of New South Wales, Sydney, Australia

**Keywords:** Antimicrobial resistance (AMR), Over-the-counter antibiotics, Standard treatment guidelines (STGs), Antibiotic dispensing, Community pharmacies, Symptomatic-treatment practices

## Abstract

**Background:**

Over-the-counter antibiotic sales in community-pharmacies significantly drive antimicrobial resistance (AMR) in low- and middle-income countries (LMICs) due to inappropriate use and early treatment discontinuation. In Bangladesh, community pharmacies, which dispense 56.6 % of antibiotics without prescriptions, serve as the first health-seeking touchpoint, yet conventional stewardship strategies often overlook these informal providers, heightening AMR risks. This study examines drug-sellers' understanding and practices towards antibiotic dispensing and compares their symptomatic-treatment practices with Bangladesh's Standard Treatment Guidelines (STGs) to understand the extent of antibiotic misuse.

**Methods:**

A cross-sectional survey in two urban and two rural areas of Bangladesh involved 120 drug-sellers from 30 randomly selected pharmacies per site. Knowledge was compared between drug-sellers with pharmacy-dispensing training and those without training, and their suggested treatments for two simulated health-symptoms—upper respiratory-tract and gastrointestinal infections—were evaluated against STGs to determine the extent of misuse.

**Results:**

Most drug-sellers were aged 41–50 years (35.0 %), with 39.2 % holding a bachelor's degree or higher, and 65.8 % having pharmacy-dispensing training. The overall knowledge score on antibiotic use and AMR was moderate at 60.2 % (5–7 out of 10), with 32.5 % scoring ≤4, indicating poor knowledge; trained drug-sellers scored significantly better (*p* = 0.008). Over half (57.5 %) were unaware of antibiotic dispensing policies, though most (75.8 %) acknowledged the link between AMR and antibiotic use. For simulated upper respiratory-tract infections, 54.2 % recommended single antibiotic-90.8 % Watch, 9.2 % Access (per WHO-AWaRe classification)-with 66.2 % of these prescriptions deviating from guidelines due to inappropriate selection or dosage. For gastrointestinal infections, 55.8 % recommended single antibiotic (40.3 % Watch, 59.7 % Access), with 82.1 % deviated from the guidelines. Additionally, 26.7 % recommended two antibiotics (51.6 % Watch, 48.4 % Access), all of which were inconsistent with guideline recommendations. For both simulated symptoms, no significant difference was observed in drug sellers' treatment practices based on their knowledge level.

**Conclusion:**

This study highlights the need for context-specific policies and regulatory measures in informal healthcare settings. While improving drug-sellers' knowledge is vital for antimicrobial stewardship in LMICs like Bangladesh, it alone is insufficient due to market competition, weak regulation, and patient-driven demand. Thus, curbing inappropriate antibiotic use at the community level requires stronger enforcement and multifaceted, context-tailored interventions—including public awareness, targeted training, and market-responsive strategies.

## 1 Introduction

Antimicrobial resistance (AMR) poses a significant global health threat,^1^ largely driven by the misuse and overuse of antimicrobials, especially in Low- and Middle-Income Countries (LMICs).^2^, ^3^, ^4^, 5., 6 In LMICs, over two-thirds of antibiotics are used for self-medication, increasing the risk of inappropriate use, including incorrect selection, low doses, short treatment durations, early discontinuation, and antibiotic sharing.^7^^,^^8^ Easy over-the-counter access to broad-spectrum antibiotics—due to poor dispensing practices and weak regulatory enforcement—further exacerbates the issue.^9^^,^^10^ In LMIC settings, community pharmacies often serve as the first point of contact for healthcare, offering both advice and medications, including antibiotics for common illnesses.^11^, ^12^, ^13^, 14. Their widespread use is driven by convenient access, flexible hours, short wait times, and affordability.^11^ In rural areas, limited access to trained professionals, poor infrastructure, and financial barriers further reinforce reliance on pharmacies as the primary source of care.^15^

In Bangladesh, there are approximately 106,919 licensed retail pharmacies, with an estimated equal number of unlicensed pharmacies.^16^ The majority of community-pharmacies are privately owned, often with limited dispensing areas, storage, and space for patient interactions.^18^ Pharmacists are required to obtain a Bachelor of Pharmacy (B.Pharm) or a Diploma in Pharmacy and pass licensing exams from the Pharmacy Council of Bangladesh.^17^ However, many drug-sellers lack proper education and licensing, with numerous unlicensed individuals in the market.^18^^,^^19^ Recent cross-sectional surveys in Bangladesh revealed that around 50 % of drug sellers lacked formal pharmacy training, having learned dispensing through apprenticeship.^16^^,^^20^ Staff qualifications vary widely; some pharmacies employ trained pharmacists, while most are staffed by individuals with little or no formal pharmacy education, failing to replicate the professional role of a pharmacist and falling short of the ideal standard of pharmacist-led care.^21^^,^^22^ In many urban and rural areas of Bangladesh, community pharmacy drug-sellers, often known as ‘village doctors,’ serve as key informal healthcare providers.^19^^,^^23^ Aggressive marketing, limited awareness among healthcare providers and patients, and poor policy enforcement drive inappropriate antibiotic use. These issues are compounded by profit motives, business sustainability in a competitive market, and a shortage of qualified pharmacists. Furthermore, weak enforcement of legal prohibitions on dispensing antibiotics without prescriptions and ineffective policy implementation exacerbate the problem.^10^ Despite legal requirements, over 50 % of outpatient antibiotics are dispensed without prescriptions in some settings, bypassing medical oversight.^12^ Furthermore, low health literacy influences patients' beliefs and behaviors, leading to increased and inappropriate antibiotic use.^24^^,^^25^

Traditional stewardship strategies, designed in high-income countries and focused on formal healthcare settings, may not be suitable for the diverse healthcare contexts of LMICs.^26^ In Bangladesh, 63 % of antibiotics are prescribed by unqualified providers, and retail pharmacies frequently dispense them without prescriptions.^27^ However, a comprehensive understanding of antibiotic dispensing practices and the factors influencing drug-sellers and informal providers remains limited.^28^ The absence of studies addressing local complexities in informal healthcare hinders evidence-based intervention development.^29^ Community-based dispensing with social and economic drivers is understudied, especially in unregulated market settings like Bangladesh.^27^ This study examines drug-sellers' understanding and analyzes their symptomatic treatment practices with antibiotics by using a novel approach of simulated patients' health symptoms. It compares their recommended treatments with Bangladesh's Standard Treatment Guidelines (STGs)^30^ to assess the extent of antibiotic misuse in symptomatic treatments.

## 2 Methods

### 2.1 Study design and settings

This cross-sectional study was conducted in four locations in Bangladesh from September 2022 to February 2023, covering two urban and two rural areas. Dhaka and Chittagong were purposively selected as urban sites due to their economic significance, while Khulna and Rangpur were randomly chosen as rural sites, followed by random selection of specific areas within these regions.

### 2.2 Data collection

Since this study focuses on antibiotic dispensing practices in community pharmacies, areas within one kilometer of general, tertiary, or specialized hospitals were excluded to avoid potential bias. Existing literature indicates that, unlike hospital-adjacent pharmacies, community pharmacies more frequently dispense antibiotics without prescriptions, and antibiotic dispensing patterns may vary between community and hospital-adjacent pharmacies.^31^, 32., 33 Pharmacy lists were obtained from local pharmacy owner associations in both urban and rural areas. After sorting the lists, 30 pharmacies were randomly selected from each site for the drug-seller survey. In cases where selected areas contained hospitals, those were omitted, and the next area on the list was chosen. The Drug-seller Survey targeted individuals managing local pharmacies, either as staff or owners, across four sites. At each selected pharmacy, one individual aged 18 or older with at least one year of drug dispensing experience was invited to participate. This resulted in a total of 120 respondents, with 30 participants from each of the two urban and two rural areas. The sample size was determined based on practical feasibility, considering available time and resources; however, efforts were made to enhance representativeness by including drug sellers from both urban and rural areas across different regions of the country. Participants included pharmacy owners who both operated the business and dispensed medicines, as well as salaried staff involved in dispensing. While formal sample size calculations were not applied, this diverse selection aimed to capture a broad range of antibiotic dispensing practices and perspectives. Nonetheless, caution is warranted when generalizing the findings beyond the study population. The data collection tool was developed based on existing literature, study objectives, and scope. It included basic demographic questions relevant to the study, such as education level, pharmacy training status, and the type and duration of training. Questions were also included to assess drug-sellers' sources of information on antibiotic dispensing and treatment, as well as their knowledge of related policies and antimicrobial resistance. Given the diversity in educational backgrounds and varying levels of training among pharmacy drug-sellers—including no training or differing training durations—the tool included key knowledge assessment questions to explore whether they understood the health risks associated with inappropriate antibiotic use, recognized their role in contributing to such misuse, were aware of its link to AMR, and had any knowledge of relevant antibiotic dispensing policies. Initially, more questions were included in the knowledge section, but piloting revealed the survey duration was too long. The number of questions was then reduced and reviewed by the research team to ensure alignment with study objectives and adequate coverage of key knowledge aspects. Knowledge was assessed using 10 questions covering topics such as antibiotic dispensing policies, antibiotic effectiveness against viruses, the relationship between antibiotic use and antimicrobial resistance (AMR), and the spread of resistant bacteria. To assess the extent of antibiotic use in drug-sellers' symptomatic-treatment practices, two simulated health scenarios—one for upper respiratory tract infections (URTIs) (Supplementary Table 1) and another for gastrointestinal infections (GIs) (Supplementary Table 2)—were presented. These scenarios were selected based on published literature from LMICs,^34^, ^35^, ^36^ particularly Bangladesh and other South Asian countries, where such conditions are among the most common reasons for non-prescription antibiotic purchases at community pharmacies. These health conditions frequently lead to non-prescription antibiotic purchases, with drug-sellers being well-acquainted with such health conditions. The researcher presented the scenarios, including patient age, weight, and symptoms, to the drug sellers and inquired about their response. If medications were suggested, the researcher asked for the specific name, dosage, and duration. Respondents could ask follow-up questions for clarification, but the data collection team was instructed not to provide additional symptom information to ensure uniformity. The survey was pre-tested with three drug-sellers in a neighboring subdistrict outside the study area. The time required for the survey was recorded, and revisions were made to simplify the questions based on the pre-test evaluation. The final survey was designed to be brief to minimize disruptions during drug-sellers' working hours. Data were collected through face-to-face interactions, and the Qualtrics survey platform was used by data collectors to input the data.

### 2.3 Data analysis

Survey data were cleaned and analyzed using STATA-15. Continuous variables such as age, education, years of experience, and pharmacy dispensing hours were categorized into ordinal groups to enhance the interpretability and communication of the results. Socio-demographic characteristics were summarized using frequencies and percentages and compared between urban and rural areas using Chi-square or Fisher exact tests (where applicable). For the knowledge Assessment, each question has been presented in a binary format, where participants responded with either ‘True/False’ or ‘Agree/Disagree.’ For each correct response, participants were awarded one point, with the total score ranging from 0 to 10. The scores were then categorized into three groups to determine the respondents' knowledge: a score of ≥8 was classified as ‘Good,’ 5–7 as ‘Moderate,’ and ≤ 4 as ‘Poor.’ We then compared the responses to each question, as well as the overall score categories, between respondents who had received training and those who had not using Chi-square or Fisher exact test. Respondents' attitude towards antibiotic use and resistance were displayed using a stacked bar graph. Statistical significance was assessed with a significance level set at *p* < 0.05.

To understand prescribers' treatment practices with antibiotics, several well-recognized guidelines are available, including the Stanford Antibiotic Guidebook,^37^ Infectious Diseases Society of America (IDSA) Guidelines,^38^ National Institute for Health and Care Excellence (NICE) Guidelines,^39^ and the WHO Essential Medicines List Antibiotic Book.^40^ However, despite the availability of these established international guidelines, local guidelines such as the Standard Treatment Guidelines (STGs) on Antibiotic Use in Common Infectious Diseases of Bangladesh, developed by the Ministry of Health and Family Welfare,^30^ were used as a benchmark. These guidelines are assumed to be more relevant and actionable, as they are tailored to Bangladesh's specific epidemiological context, healthcare practices, and resource constraints, aiming to ensure the appropriate use of antibiotics by providing clear treatment recommendations for common infections. During the analysis, STGs were selected to assess the extent of inappropriate antibiotic use, particularly regarding course regimens, in two simulated health scenarios involving drug-sellers' empirical symptomatic-treatment practices. For this analysis, an analysis protocol was developed. The antibiotic/s recommended by drug-sellers, along with dosages for each health-scenario, were then incorporated into three independent variables—‘Antibiotic/s selection’, ‘Recommended duration’, and ‘Recommended daily doses’. Another three independent variables were incorporated from the STGs— ‘Recommended Antibiotic/s’, ‘Standard recommended duration’, and ‘Standard recommended daily doses’ for each health-scenario. The drug-sellers' recommended antibiotics, along with dosages, were then compared with the STGs-recommended antibiotics and dosages for each health-scenario. If the drug-sellers' recommended antibiotics and dosages aligned with the STGs' recommendations, the dependent variable, ‘Antibiotic utilization status,’ was classified as ‘Aligned’. If any of the independent variables did not match (indicating underutilization or overutilization) with the STGs, it was classified as ‘Deviated’. Additionally, the distribution of recommended antibiotic/s was analyzed according to the World Health Organization (WHO) AWaRe (Access, Watch, Reserve) classifications, a tool designed to guide the appropriate use of antibiotics and help combat AMR,^41^ with a particular focus on the proportions in the ‘Access’ and ‘Watch’ categories. These findings were presented in two tables corresponding to the two simulated health-scenario. Similar analytical methods have been applied in previous studies to assess adherence to antibiotic use among patients who obtained antibiotics with or without prescriptions from community pharmacies.^42^

## 3 Results

### 3.1 Demographic characteristics

Table 1 summarizes the characteristics of drug-sellers from rural and urban areas. The majority are male (99.2 %), with half aged 40 or younger. Approximately 39.2 % have completed a bachelor's degree or higher, and 35 % have attained higher secondary education. A significant portion of drug-sellers has extensive pharmacy experience, with 26.7 % working for 11 to 20 years. Among them, 18.3 % are known as village-doctors for their long-term practice in symptomatic treatment, despite lacking medical degrees. Additionally, 65.8 % of drug-sellers have received training in pharmacy dispensing, primarily through a 6-month certificate program (36.7 %) or a pharmacy technician certificate ‘Grade-C' course (15.6 %). Their main sources of information on antibiotic use (indications and dosage) are registered physicians' prescriptions (85.8 %) and pharmaceutical company sales representatives (75.0 %).

**Table 1:** Characteristics of the drug-sellers.

Characteristics	Categories	Total	Rural	Urban	*P*-value
		n/120 (%)	n/60 (%)	n/60 (%)	
Sex	Male	119 (99.2)	59 (98.3)	60 (100.0)	0.315
Age (years)	20–30	31 (25.8)	16 (26.7)	15 (25.0)	0.127
	31–40	31 (25.8)	10 (16.7)	21 (35.0)	
	41–50	42 (35.0)	25 (41.7)	17 (28.3)	
	51 and above	16 (13.3)	9 (15.0)	7 (11.7)	
Education	≤ Grade 5 (primary education)	4 (3.33)	2 (3.3)	2 (3.3)	0.131⁎
	≤ Grade 10 (S.S.C.)	27 (22.5)	15 (25.0)	12 (20.0)	
	Completed grade 12 (H.S.C.)	42 (35.0)	15 (25.0)	27 (45.0)	
	Completed a bachelor's degree or higher	47 (39.2)	28 (46.7)	19 (31.7)	
Pharmacy work experience (years)	≤ 2	18 (15.0)	12 (20.0)	6 (10.0)	0.145
	3 to 5	24 (20.0)	7 (11.7)	17 (28.3)	
	6 to 10	24 (20.0)	14 (23.3)	10 (16.7)	
	11 to 20	32 (26.7)	16 (26.7)	16 (26.7)	
	21 years	22 (18.3)	11 (18.3)	11 (18.3)	
Known as ‘Village doctor’	Yes	22 (18.3)	10 (16.7)	12 (20.0)	0.637
Received pharmacy training	Yes	79 (65.8)	38 (63.3)	41 (68.3)	0.564

	Of those with training	n/79 (%)	n/38 (%)	n/41 (%)	
Length of training	Pharmacy technician certificate course (Grade-C)	36 (15.6)	17 (44.7)	19 (46.3)	0.232⁎
	6 months certificate course	29 (36.7)	11 (28.9)	18 (43.9)	
	1 year certificate course	8 (10.1)	6 (15.8)	2 (4.9)	
	Diploma-in-Pharmacy (Grade B, 3 year)	6 (7.6)	4 (10.5)	2 (4.9)	

	Multiple responses	266/120 (%)	123/60 (%)	143/60 (%)	
Sources of knowledge on antibiotic use^1^	Physicians' prescription	103 (85.8)	50 (83.3)	53 (88.3)	0.132⁎
	Pharma. representatives	90 (75.0)	40 (66.7)	50 (83.3)	
	Own experience in this trade	64 (52.3)	28 (46.7)	36 (60.0)	
	Others	9 (7.5)	5 (8.3)	4 (6.7)	

⁎ P-Value for Chi-square (χ2) test, P-Value for Fisher's exact test.

1 Multiple sources of knowledge on antibiotic use were incorporated.

### 3.2 Knowledge, perceptions and attitude on antibiotic use and resistance

The overall knowledge score of drug-sellers on antibiotic use and resistance was moderate (5–7 out of 10) at 60.2 %, with 32.5 % scoring ≤4, indicating poor knowledge (Table 2). Individuals who received any duration of pharmacy training exhibited slightly better scores, with a statistically significant difference (*p* = 0.008) according to Fisher's exact test. Over half (57.5 %) of respondents were unaware of specific antibiotic dispensing policies in the country, with no significant difference between trained and untrained individuals. Most respondents (75.8 %) showed awareness of antimicrobial resistance (AMR) and recognized the link between antibiotic dispensing (role of drug-sellers) and AMR. However, 33.3 % believed that antibiotics are effective against viruses, and 14.2 % thought the same about colds and flu. Surprisingly, 50.8 % felt that treatment with antibiotics, regardless of infection status, would not increase the risk of AMR; however, those who received training demonstrated significantly better knowledge on this issue.

**Table 2:** Knowledge and perceptions on antibiotic use and resistance.

Knowledge indicators	Categories	Total	With training	Without training	P-value
		n/120 (%)	n/79 (%)	n/41 (%)	
Our country has a policy on antibiotic dispensing	Disagree	69 (57.5)	44 (55.7)	25 (61.0)	0.579
I am familiar with AMR	Yes	91 (75.8)	66 (83.5)	25 (61.0)	0.006
There is a connection between my antibiotic dispensing and AMR	True	91 (75.8)	63 (79.8)	28 (68.3)	0.165
Antibiotics are effective against viruses	True	40 (33.3)	26 (32.9)	14 (34.2)	0.892
Antibiotics are effective against cold and flu	True	17 (14.2)	12 (15.2)	5 (12.2)	0.655
Unnecessary use of antibiotics makes them ineffective	True	106 (88.3)	71 (89.9)	35 (85.4)	0.466
Taking antibiotics has associated side effects or risks	True	100 (83.3)	70 (88.6)	30 (73.2)	0.031
Every individual treated with antibiotics faces an increased risk of antimicrobial resistance	False	61 (50.8)	31 (39.2)	30 (73.2)	<0.001
Antibiotic-resistant bacteria can spread between people	False	89 (74.2)	56 (70.9)	33 (80.5)	0.254
Healthy people can carry antibiotic-resistant bacteria	False	89 (74.2)	58 (73.4)	31 (75.6)	0.795
**Overall knowledge score** (Out of 10)	Good (≥8)	10 (8.5)	8 (10.1)	2 (5.1)	0.008⁎
	Moderate (5–7)	71 (60.2)	53 (67.1)	18 (46.2)	
	Poor (≤4)	39 (32.5)	18 (22.8)	21 (51.2)	

⁎ P-Value for Chi-square (χ2) test, P-value for Fisher's exact test.

**Fig. 1:**
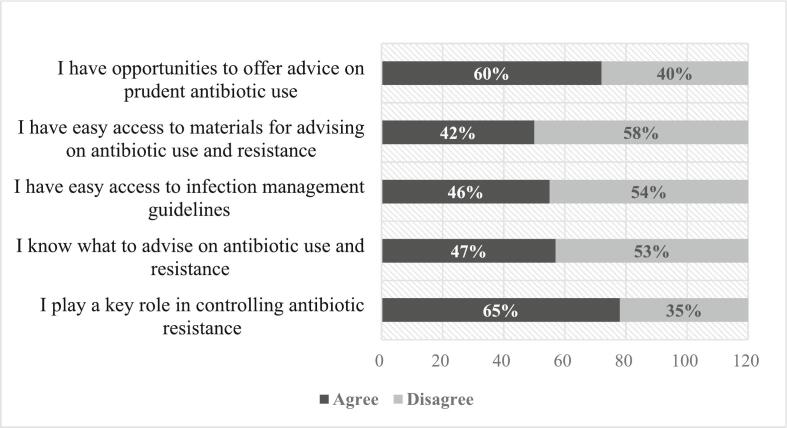
Attitudes related to antibiotic use and resistance.

Fig. 1 summarizes drug-sellers' attitudes towards antibiotic use and resistance. The majority of respondents (60 %) agreed that as drug-sellers, they have the opportunity to advise on prudent antibiotic use, while 65 % believe they can play a key role in controlling antibiotic resistance (Fig. 1). However, many reported a lack of access to essential resources: 58 % indicated they do not have easy access to materials for advising on antibiotics, 54 % lack access to infection management guidelines, and 53 % are unsure about what advice to provide regarding antibiotic use and resistance.

### 3.3 Drug-sellers' practices in symptomatic-treatment with antibiotics

#### 3.3.1 Simulated respiratory-tract infections scenario

In response to the first simulated scenario (Table 3), which resembles typical upper respiratory-tract infections, only 5 % of drug-sellers would refer the patient to a registered physician for diagnosis and treatment. Meanwhile, 40.8% would recommend non-antibiotic medications, primarily antihistamines and non-steroidal anti-inflammatory drugs. The majority (54.2 %) would prescribe antibiotics alongside non-antibiotic medications. Notably, 90.8 % of the prescribed antibiotics were classified as ‘Watch’ according to WHO-AWaRe classifications. We assessed the extent of deviation in antibiotics prescribed by drug-sellers for upper respiratory-tract infections from STGs. Our findings indicate that 66.2 % of antibiotic recommendations were deviated from STGs, either in terms of incorrect antibiotic selection or improper dosage. According to STGs, antibiotics should only be used for specific conditions and microorganisms by a registered physician. Symptomatic treatment practices for upper respiratory tract infections were also assessed based on drug sellers' knowledge levels. However, no significant differences were found between those with good/moderate and poor knowledge scores.

**Table 3:** Drug-sellers' responses to simulated upper respiratory-tract infections scenario.

Drug-sellers' response	n/120 (%)	Based on knowledge score⁎	
		Good/moderate n/81 (%)	Poor n/39 (%)
Refused to provide treatment and refer to physicians	6 (5.0)	3 (3.7)	3 (7.7)
Recommended non-antibiotic medications	49 (40.8)	34 (42.0)	15 (38.5)
Recommended one antibiotic (with or without other non-antibiotic medications)	65 (54.2)	44 (54.3)	21 (53.8)


Details of the recommended antibiotic				
WHO-AWaRe classifications		Antibiotic name (generic)	n/65 (%)	Deviation from STGs⁎⁎ n/65 (%)
Access	9.2 %	Amoxicillin	6 (9.2)	4 (6.2)
Watch	90.8 %	Azithromycin	45 (69.2)	29 (44.6)
		Cefixime	8 (12.3)	4 (6.2)
		Cefuroxime	4 (6.2)	4 (6.2)
		Ciprofloxacin	2 (3.1)	2 (3.0)
				Total- 43 (66.2)

⁎ Based on the drug sellers' knowledge scores in Table 2.

⁎⁎ Deviation from STGs' refers to any instance where the recommended antibiotic treatment does not align with the Bangladesh Standard Treatment Guidelines, including incorrect drug selection, dosage, or duration.

#### 3.3.2 Simulated gastrointestinal infection scenarios

In response to the second simulated-scenario (Table 4), reflecting a typical gastrointestinal infection with watery stools for three days, 5 % of drug-sellers would not provide symptomatic-treatment and would refer the patient to a registered physician, 12.5 % recommended for non-antibiotic treatments, primarily antispasmodics, antiparasitic, and antidiarrheals along with oral rehydration therapy (ORT). Just over half (55.8 %) prescribed one antibiotic alongside ORT and other non-antibiotic medications. According to WHO-AWaRe classifications, 59.7 % of the recommended antibiotics were categorized as ‘Access’ and 40.3 % as ‘Watch’. Of the recommended single antibiotics, 82.1 % deviated from STGs in terms of selection, dosage, or duration. Additionally, 26.7 % of drug-sellers recommended two antibiotics along with non-antibiotic medications and ORT. These combinations predominantly included one ‘Access’ antibiotic in 48.4 % of instances and one ‘Watch’ antibiotic in 51.6 % of instances. As treating such conditions with two antibiotics is not found in the STGs, the recommended antibiotics were considered fully deviated in terms of antibiotic choice, combination, and recommended dosage. Drug sellers' symptomatic treatment practices for gastrointestinal infections were also evaluated according to their knowledge levels, but no significant differences were observed between those with good/moderate and poor knowledge scores.

**Table 4:** Drug-sellers' responses to simulated gastrointestinal infection scenarios.

Drug-sellers' response	n/120 (%)	Based on knowledge score⁎	
		Good/moderate n/81 (%)	Poor n/39 (%)
Refused to provide treatment and refer to physicians	6 (5.0)	2 (2.5)	4 (10.3)
Recommended non-antibiotic medications	15 (12.5)	12 (14.8)	3 (7.7)
Recommended one antibiotic (with or without other non-antibiotic medications)	67 (55.8)	43 (53.1)	24 (61.5)
Recommended two antibiotics (with or without other non-antibiotic medications)	32 (26.7)	24 (29.6)	8 (20.5)


Details of the recommended one antibiotic				
WHO-AWaRe classifications		Antibiotic name (generic)	n/67 (%)	Deviation from STGs⁎⁎ n/67 (%)
Access	59.7 %	Metronidazole	40 (59.7)	36 (53.7)
Watch	40.3 %	Ciprofloxacin	20 (29.8)	16 (23.9)
		Azithromycin	4 (6.0)	1 (1.5)
		Cefixime	3 (4.5)	2 (3.0)
				Total- 55 (82.1)


Details of the recommended two antibiotics				
WHO-AWaRe classifications	Antibiotics name (generic)		n/32 (%)	Deviation from STGs⁎⁎ n/32 (%)
Access (48.4 %)	Metronidazole (Access)	Ciprofloxacin (Watch)	24 (75.0)	24 (75.0)
	Metronidazole (Access)	Azithromycin (Watch)	5 (15.6)	5 (15.6)
Watch (51.6 %)	Metronidazole (Access)	Cefuroxime (Watch)	1 (3.1)	1 (3.1)
	Metronidazole (Access)	Chlortetracycline (Watch)	1 (3.1)	1 (3.1)
	Azithromycin (Watch)	Ciprofloxacin (Watch)	1 (3.1)	1 (3.1)
				Total- 32 (100.0)

⁎ Based on the drug sellers' knowledge scores in Table 2.

⁎⁎ Deviation from STGs' refers to any instance where the recommended antibiotic treatment does not align with the Bangladesh Standard Treatment Guidelines, including incorrect drug selection, dosage, or duration.

## 4 Discussion

This study highlights that community pharmacy drug-sellers in Bangladesh frequently dispense antibiotics without prescriptions, violating the National Drug Policy,^17^^,^^43^ which mandates a registered physician's prescription for antibiotics and other prescription drugs.^44^ Drug-sellers also empirically prescribe antibiotics for symptomatic treatments, often with inappropriate choices or dosages, contributing to misuse and antimicrobial resistance (AMR). This is compounded by their lack of formal training and limited knowledge of safe dispensing practices and the risks associated with misuse. Despite regulatory requirements, in Bangladesh, retail drug shops often serve as the primary and sole healthcare source for many patients, with over 80 % of the population depending on untrained or inadequately trained village-doctors and drug shop retailers, aligning with other studies in Bangladesh and LMICs.^16^^,^^45^ Similar trends are observed across other low- and middle-income countries (LMICs).^17^^,^^43^^,^^46^ A study in Bangladesh reported that, despite having no formal training, pharmacy drug sellers were twice as likely as physicians to deliver treatment to the public.^47^ The irrational use of antibiotics is driven by complex interactions among healthcare providers, patients, and caregivers, shaped by socio-economic factors and health-seeking behaviors, as highlighted in recent studies.^48^^,^^49^ Patient expectations, lack of awareness, and aggressive marketing by pharmaceutical companies further drive inappropriate antibiotic use.^10^

Findings show that a significant proportion of drug-sellers recommended Watch group antibiotics, with widespread deviations from standard treatment guidelines for upper respiratory and gastrointestinal infections, including inappropriate combinations, durations, and frequencies—reflecting pervasive misuse. The WHO's AWaRe classification advises that 60 % of antibiotic use should come from the Access group to combat resistance.^50^ Yet, a recent Bangladeshi survey found that 56.6 % of antibiotics were dispensed without prescriptions, with 73.5 % from the Watch group and only 23.1 % from the Access group,^45^ reflecting a concerning trend. Patients often cannot differentiate between qualified and unqualified providers, perceiving drug-sellers—often referred to as ‘village doctors’—as legitimate healthcare sources, play a significant role as informal healthcare providers and commonly treat patients with antibiotics.^17^^,^^19^ According to the National Drug Policy of Bangladesh, drug-sellers are not authorized to recommend antibiotics to patients^43^; however, antibiotic prescription is permitted for registered physicians and, in specific cases, certain first-line primary healthcare providers in government facilities, such as Sub-Assistant Community Medical Officers (SACMOs) and Community Health Care Providers (CHCPs), who are authorized to prescribe a limited range of antibiotics.^17^

According to national regulations, a Grade C pharmacist—trained through a 12-week basic course—is required for dispensing drugs.^51^ However, this study found 34.2 % of drug-sellers lacked this training, aligning with other studies indicating that 49 % of salespeople have no formal training but still prescribe and dispense antibiotics.^16^^,^^17^ Drug-sellers primarily depend on prescriptions from other doctors and information from pharmaceutical sales representatives, as observed in this study. Across South Asia and LMICs, pharmaceutical companies significantly influence prescribing through promotional incentives and credit-based sales, often encouraging polypharmacy and overuse of expensive or unnecessary antibiotics.^17^^,^^27^^,^^52^ This promotional environment emphasizes benefits while minimizing risks, as noted in several reviews.^46^^,^52., 53, 54 In this study, more than half of the drug-sellers were unaware of prescription requirements and demonstrated only moderate knowledge of antibiotics and AMR. This aligns with findings from Sri Lanka, where limited understanding led to widespread OTC antibiotic sales.^10^ Despite some awareness of the link between unnecessary antibiotic use and AMR, financial pressures and competitive market dynamics compel drug-sellers to continue OTC sales.^17^ Many drug-sellers also misunderstand antibiotic efficacy against viral infections, such as colds and flu, contributing to inappropriate usage patterns common in Southeast Asia and LMICs.^55^ Proposed regulatory actions in Bangladesh—including fines and imprisonment—seek to deter illegal dispensing,^56^ but enforcement remains weak due to systemic issues: poor infrastructure, limited trained personnel, underfunding, and complex monitoring processes.^16^^,^^17^^,^^57^^,^^58^

Prescription-only regulations can reduce OTC sales, but their effectiveness is limited without rigorous evaluation and tailored interventions.^59^ The competitive market further undermines compliance, as drug-sellers fear income loss.^17^^,^^45^^,^^60^ In the highly competitive market in Bangladesh, there are no restrictions on the location of pharmacy premises. As such with about 7.7 pharmacies per 10,000 people.^61^ Despite frequently recommending full courses to enhance their reputation and profits, customers sometimes refuse, considering them unnecessary.^17^ Drug-sellers often avoid insisting on prescriptions or advising against partial course antibiotics to prevent losing customers to competitors.^10^^,^^17^ The WHO report on antibiotic packaging and stewardship in Bangladesh highlights enforcement challenges in curbing OTC antibiotic sales due to 86 local manufacturers supplying 98 % of the country's pharmaceuticals and intense competition among numerous pharmacies. Additionally, limited public awareness of antibiotics and AMR continues to drive inappropriate use.^62^ While educational campaigns and pharmacist training improve knowledge and consultation practices,^63^^,^^64^ this study found no significant differences in antibiotic dispensing or appropriateness of symptomatic treatment were observed among drug-sellers regardless of knowledge level, indicating that knowledge alone does not translate into appropriate practice. This suggests that without addressing business revenue and reducing customer demand for OTC antibiotics, knowledge enhancement is insufficient.^19^^,^^45^^,^^65^^,^^66^ A recent study stresses the need to consider economic impacts on pharmacy businesses when implementing antimicrobial stewardship programs (ASPs).^67^ As integral actors in LMIC health systems, community pharmacies can support ASPs by reinforcing prescriptions, addressing non-adherence, and improving patient understanding of antibiotic use.^13^^,^^14^^,^^68^ Future research should further examine commercial determinants of health and antibiotic marketing, balancing business interests with public health goals. This includes developing politically feasible strategies, enhancing pharmacy training, addressing regulatory gaps, evaluating the economic impact of ASPs, and exploring models to stabilize pharmacy incomes while promoting responsible antibiotic use.^69^^,^^70^

### 4.1 Limitations

Despite efforts to minimize gaps, certain limitations remain. To capture antibiotic dispensing practices in community settings and reduce potential bias, pharmacies within one kilometer of general, tertiary, or specialized healthcare facilities were excluded. Hospital-adjacent pharmacies often report higher antibiotic sales due to severe illnesses and surgical cases, whereas this study focused on the knowledge and practices of drug sellers in community pharmacy settings. As a result, the findings reflect practices of community pharmacy drug sellers and may not represent those near hospitals. The survey explored drug-sellers' training, knowledge, and practices; however, the scope of knowledge- and practice-related questions was limited to maintain a reasonable survey duration. Basic questions were included to assess understanding of AMR, its link to unnecessary antibiotic use, and awareness of antibiotic dispensing regulations. Since drug-sellers' responses were self-reported in the presence of a researcher, they may have been influenced by the Hawthorne Effect or social desirability bias. To mitigate this, two simulated health scenarios with patient details were introduced; however, bias may still exist as participants were aware they were being evaluated. The Standard Treatment Guidelines (STGs) for Antibiotic Use in Common Infectious Diseases of Bangladesh were used as a benchmark to assess the appropriateness of antibiotic selection, dosage, frequency, and duration recommended for each simulated health condition. While both national policy and STGs prohibit antibiotic dispensing without a registered healthcare professional's prescription, the study aimed to examine the extent and nature of antibiotic misuse for common symptoms that prompt people to seek treatment from community pharmacies—an area underexplored in current literature. Comparisons were limited to the national STGs, potentially excluding other relevant guidelines. Thus, findings should be interpreted with caution, highlighting the need for further research in similar contexts.

## 5 Conclusion

In resource-constrained community contexts, irrational antibiotic use stems from complex interactions among healthcare providers, patients, and caregivers, shaped by socio-economic conditions and health-seeking behaviors. The study highlights inadequate mandatory training, significant knowledge gaps, and unauthorized symptomatic antibiotic use that contradicts standard treatment guidelines, emphasizing the need for context-specific policies and regulations in informal healthcare settings. Effective stewardship in LMICs requires multifaceted strategies, including mandatory dispensing training as a prerequisite for pharmacy registration, context-specific regulations, robust monitoring through random inspections, and complaint mechanisms such as local hotlines for non-compliance.

Regulatory changes, such as color-coding antibiotic packaging, could raise public awareness and promote informed decision-making. While WHO recommends these measures to support AMS in Bangladesh, challenges persist due to limited awareness of antibiotics and AMR, the large number of pharmacies (∼202,500), and numerous local manufacturers, complicating enforcement by the DGDA. Although pharmaceutical companies generally support these changes for public health, the added costs may burden smaller manufacturers. Logistical adjustments and potential resistance from pharmacy owners will require clear communication and appropriate incentives. Knowledge improvement and law enforcement alone are insufficient to reduce non-prescription antibiotic dispensing; understanding market dynamics and offering practical guidelines to help pharmacy owners balance business sustainability with policy adherence is essential. Further research should explore the feasibility and impact of enforcing prescription-only antibiotic sales, as well as full-course packaging in Bangladesh's competitive pharmacy market. Understanding motivations behind self-medication and implementing tailored approaches for diverse socio-economic contexts are key to improving practices and policy outcomes. Community pharmacies, as part of the informal health system, can contribute to antimicrobial stewardship by reinforcing physicians' advice and promoting appropriate antibiotic use through targeted training, motivation, and support.

## Data sharing

The datasets utilized in this study are accessible from the corresponding author upon reasonable request, in accordance with the data sharing policies of the University of New South Wales and BRAC James P. Grant School of Public Health. Supplementary files, including supporting data and summaries, are available.

## Ethics approval

The study was approved by the University of New South Wales (UNSW) Human Research Ethics Committee (approval reference- HC220360) and the Institutional Review Board (IRB) of BRAC James P. Grant School of Public Health in Bangladesh (IRB protocol number- IRB-22 September’22–037). Before the survey, the research team informed pharmacy owners about the study objectives and their role, assuring them that the survey would minimally impact sales and not affect the pharmacies' reputation or legal standing. Written consent forms in the local language were provided, and respondents signed before starting the survey, with each participant assigned a unique, non-identifiable code.

## Funding

This study is a component of the International Society of Antimicrobial Chemotherapy (ISAC)-funded project grant 2021 titled “ Practices of antibiotic consumption and dispensing in the middle and low-income people in Bangladesh: a potential contributor to the emergence of antimicrobial resistance in the community”. The funders had no role in the design, execution, or interpretation of the study.

## Author's contributor

HS, RLW, MS, and AAM led the conception and design of the study. MA also contributed to this process. HS, RLW, AAM, MS, MA, and MSI were involved in securing funding for the project. HS, RLW, MS, AAM, MA, MSI, AK, and ZA contributed to developing the methodology. AAM, HS, MS, RLW, and ATM ensure the availability of the resources for project administration and consistently follow up on the implementation. AAM, HS, RLW, MS, and ATM were involved in the investigation at the primary and secondary levels. Data curation was jointly undertaken by AAM, RLW, AK, ATM, and HS. Data analysis, spanning primary and secondary levels, engaged AAM, AK, ZA, RLW, and HS, with AK and AAM utilizing software tools for analysis. AAM, RLW, AK, MS, MA, MSI, ZA, and HS were involved in data validations. AAM, RLW, AK, and HS were involved in data visualization. AAM was primarily responsible for preparing the primary draft and finalizing the final draft of this manuscript and others provided their inputs.

## Declaration on the use of AI assisted tools

During the preparation of this manuscript, AI-assisted tools were used limitedly for language editing and fixing grammatical errors.

## CRediT authorship contribution statement

**Abdullah Al Masud:** Writing – review & editing, Writing – original draft, Visualization, Validation, Supervision, Software, Resources, Project administration, Methodology, Investigation, Funding acquisition, Formal analysis, Data curation, Conceptualization. **Ramesh Lahiru Walpola:** Writing – review & editing, Visualization, Validation, Supervision, Resources, Methodology, Investigation, Funding acquisition, Conceptualization. **Malabika Sarker:** Writing – review & editing, Visualization, Validation, Supervision, Project administration, Methodology, Investigation, Funding acquisition, Conceptualization. **Muhammad Asaduzzaman:** Writing – review & editing, Visualization, Validation, Methodology, Investigation, Funding acquisition, Conceptualization. **Md. Saiful Islam:** Writing – review & editing, Supervision, Methodology, Funding acquisition, Conceptualization. **Ayesha Tasnim Mostafa:** Resources, Project administration, Data curation. **Zubair Akhtar:** Writing – review & editing, Formal analysis. **Alamgir Kabir:** Writing – review & editing, Visualization, Validation, Supervision, Software, Methodology, Investigation, Formal analysis. **Holly Seale:** Writing – review & editing, Visualization, Validation, Supervision, Resources, Project administration, Methodology, Investigation, Funding acquisition, Formal analysis, Conceptualization.

## Declaration of competing interest

The authors declare no conflicts of interest regarding the presented research. Any potential competing interests have been acknowledged and managed to maintain the study's integrity and impartiality.

## Appendix A Supplementary data

Supplementary material: Data collection format for assessing drug-sellers' symptomatic treatment practices using simulated scenarios Image 1
