# Supplementary material for: Antibiotic dispensing practices in community pharmacies: Implications for antimicrobial stewardship in resource-constrained settings
Source: Explor Res Clin Soc Pharm. 2025 Apr 22;19:100606. doi: 10.1016/j.rcsop.2025.100606 (PMC12142531; doi:10.1016/j.rcsop.2025.100606)
Supplement: Supplementary file 1 — Supplementary material: Data collection format for assessing drug-sellers' symptomatic treatment practices using simulated scenarios [file mmc1.docx]

**Supplementary table 1: Upper respiratory-tract infections**

**Assessment of drug-sellers' symptomatic-treatment practices using simulated health symptoms**

| A 21-year-old man (Weight 68 Kg), having a mild fever (100°C) for 2 days with a 3-day cold, productive cough, and nose congestion has come to you and asked for medicine from you, what will you suggest? Please describe the suggestions that you will provide to him. | | | | | |
| --- | --- | --- | --- | --- | --- |
| Response: | | | | | |
| 1. I will not give him any treatment and will suggest visiting a registered physician | | | | | |
| 1. Based on his symptoms, I will provide treatment | | | | | |
| **Medications** | | **Medication name** | **Dose (mg.)** | **Frequency** | **Duration** |
| Medication 1 | ☐ Antibiotic  ☐ Non-antibiotic  ☐ Other (Specify) |  |  | ☐ 6-hourly  ☐ 8-hourly  ☐ 12-hourly  ☐ 24-hourly  ☐ Other (Specify) |  |
| Medication 2 | ☐ Antibiotic  ☐ Non-antibiotic  ☐ Other (Specify) |  |  | ☐ 6-hourly  ☐ 8-hourly  ☐ 12-hourly  ☐ 24-hourly  ☐ Other (Specify) |  |
| Medication 3 | ☐ Antibiotic  ☐ Non-antibiotic  ☐ Other (Specify) |  |  | ☐ 6-hourly  ☐ 8-hourly  ☐ 12-hourly  ☐ 24-hourly  ☐ Other (Specify) |  |
| Medication 4 | ☐ Antibiotic  ☐ Non-antibiotic  ☐ Other (Specify) |  |  | ☐ 6-hourly  ☐ 8-hourly  ☐ 12-hourly  ☐ 24-hourly  ☐ Other (Specify) |  |
| Medication 5 | ☐ Antibiotic  ☐ Non-antibiotic  ☐ Other (Specify) |  |  | ☐ 6-hourly  ☐ 8-hourly  ☐ 12-hourly  ☐ 24-hourly  ☐ Other (Specify) |  |

Note: Respondents may ask follow-up questions for further clarification, but no additional information about the symptoms will be provided to ensure uniformity.

**Supplementary table 2: Gastrointestinal infection**

**Assessment of drug-sellers' symptomatic-treatment practices using simulated health symptoms**

| A 25-year-old woman (Weight 56 Kg), has been suffering from watery diarrhea with 10 to 12 times watery stool frequency for 3 days and abdominal pain has come to you and asked for medicine from you, what will you suggest? Please describe the suggestions that you will provide to her. | | | | | |
| --- | --- | --- | --- | --- | --- |
| Response: | | | | | |
| 1. I will not give him any treatment and will suggest visiting a registered physician | | | | | |
| 1. Based on his symptoms, I will provide treatment | | | | | |
| **Medications** | | **Medication name** | **Dose (mg.)** | **Frequency** | **Duration** |
| Medication 1 | ☐ Antibiotic  ☐ Non-antibiotic  ☐ Other (Specify) |  |  | ☐ 6-hourly  ☐ 8-hourly  ☐ 12-hourly  ☐ 24-hourly  ☐ Other (Specify) |  |
| Medication 2 | ☐ Antibiotic  ☐ Non-antibiotic  ☐ Other (Specify) |  |  | ☐ 6-hourly  ☐ 8-hourly  ☐ 12-hourly  ☐ 24-hourly  ☐ Other (Specify) |  |
| Medication 3 | ☐ Antibiotic  ☐ Non-antibiotic  ☐ Other (Specify) |  |  | ☐ 6-hourly  ☐ 8-hourly  ☐ 12-hourly  ☐ 24-hourly  ☐ Other (Specify) |  |
| Medication 4 | ☐ Antibiotic  ☐ Non-antibiotic  ☐ Other (Specify) |  |  | ☐ 6-hourly  ☐ 8-hourly  ☐ 12-hourly  ☐ 24-hourly  ☐ Other (Specify) |  |
| Medication 5 | ☐ Antibiotic  ☐ Non-antibiotic  ☐ Other (Specify) |  |  | ☐ 6-hourly  ☐ 8-hourly  ☐ 12-hourly  ☐ 24-hourly  ☐ Other (Specify) |  |

Note: Respondents may ask follow-up questions for further clarification, but no additional information about the symptoms will be provided to ensure uniformity.
